# Endoscopic closure of a malignant entero-vaginal fistula using a cardiac septal occluder device

**DOI:** 10.1055/a-2872-7115

**Published:** 2026-05-29

**Authors:** Patricia Mester, Can-Martin Sag, Vlad Pavel, Martina Müller, Hans-Jürgen Schlitt, Matthias Hornung, Arne Kandulski

**Affiliations:** 1 Department of Internal Medicine I, Gastroenterology, Hepatology, Endocrinology, Rheumatology, and Infectious Diseases 39070 University Hospital RegensburgRegensburgBYGermany; 2 Department of Internal Medicine II, Cardiology 39070 University Hospital RegensburgRegensburgBYGermany; 3 Surgery 39070 University Hospital RegensburgRegensburgBYGermany

Enterovaginal fistulas (EVFs) are pathological communications between the small bowel and the vagina 1 . Malignancy is the most common underlying cause 2 . Diagnosis is usually radiologic and/or endoscopic [3. Management includes minimally invasive endoscopy or surgery 1 . Cardiac septal occluder (CSO) devices, originally developed for transcatheter closure of atrial and ventricular septal defects 3 , have only recently been investigated as a treatment option for gastrointestinal fistulas 4 .

Here, we report the case of a 44-year-old woman with advanced pseudomyxoma peritonei who had previously undergone multiple abdominal operations, including small- and large-bowel resections with end-to-end ileostomy and descending colostomy. At the current admission, she presented with feculent vaginal discharge. Computed tomography (CT) demonstrated an enterovaginal fistula. The case was interdisciplinary discussed, and in view of her extensive surgical history, endoscopic closure of the fistula was selected as the preferred treatment approach.

**Fig. 1:**
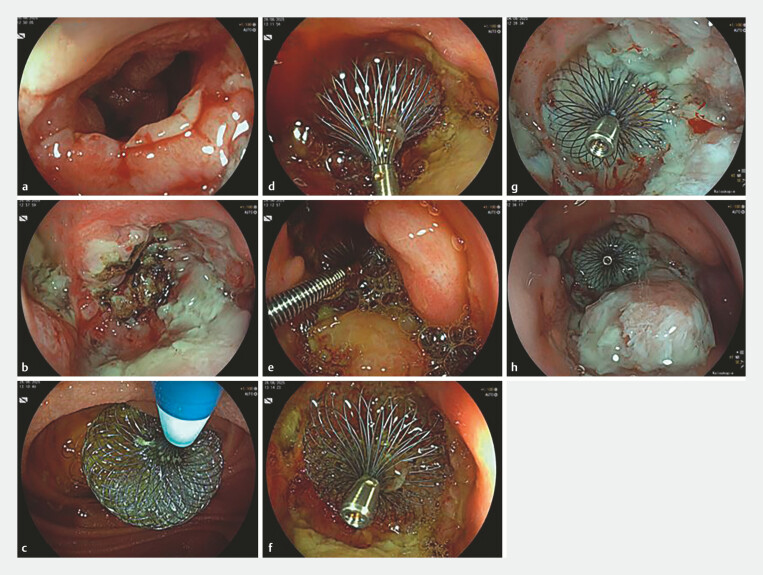
Transvaginal endoscopic treatment of an enterovaginal fistula (EVF) using a cardiac septal occluder (CSO) device. **a** Transvaginal endoscopic visualization of the EVF. **b** Argon plasma coagulation of the fistula tract to induce granulation tissue (forced APC, 30 W; Erbe Elektromedizin, Tübingen, Germany). **c** Placement of the CSO with release of the distal flare in the small bowel. **d,e** Retraction of the CSO from the small bowel into the vaginal lumen and release of the proximal flare, resulting in closure of the EVF. **f** Complete deployment of the CSO. **g,h** One-week surveillance endoscopy demonstrating granulation tissue around the CSO.

The transvaginal approach was deemed most appropriate. Transvaginal application of an over-the-scope clip (OTSC; Ovesco, Tübingen, Germany) was not feasible because of marked fibrotic changes. Endoscopic suturing has also been taken into consideration. Because of the large defect, CSO was considered a better option in this case. First, the margins of the EVF were cauterized using argon plasma coagulation (forced APC; Erbe, Tübingen, Germany) ( Fig. 1 **a,b** ). A guidewire (Amplatz Super Stiff, 0.35 F, Boston Scientific Corp.) was then advanced across the fistula into the small-bowel lumen via the endoscope. Of note, special endoscopic devices are not necessary because this type of guidewire can be inserted through a standard endoscope. However, the CSO device could also be introduced through the working channel of the endoscope. Nevertheless, if this approach is chosen, the diameter and length of both the endoscope and the CSO must be taken into consideration because various CSO devices are available. Afterwards the endoscope was retrieved and a 10-mm Amplatzer septal occluder device (Abbott Cardiovascular) preloaded in a 6F introducer sheath was introduced under endoscopic guidance and deployed ( Fig. 1 **c-f** ). After deploying the proximal flange, the distal one was deployed in the vaginal remanent surface, fully occluding the defect. The patient was observed for 24 hours, during which no procedure-related adverse events occurred. One week after the intervention, follow-up endoscopy demonstrated the CSO in stable position with surrounding granulation tissue ( Fig. 1 **g,h** ) and the patient reported complete resolution of vaginal discharge. Three months after the intervention, the patient denied any vaginal discharge and presented with improved quality of life. This case illustrates that endoscopic placement of occluder devices can be a valuable addition to the therapeutic armamentarium for enterovaginal fistulas, particularly in patients with malignancy and complex postoperative abdominal anatomy, in whom conventional surgical options are limited ( Video 1 ).

Transvaginal endoscopic treatment of an enterovaginal fistula (EVF) using a cardiac septal occluder (CSO) device.Video 1
